# Neuronal substance P-driven MRGPRX2-dependent mast cell degranulation products differentially promote vascular permeability

**DOI:** 10.3389/fimmu.2024.1477072

**Published:** 2024-11-21

**Authors:** Masakazu Nagamine, Ayako Kaitani, Kumi Izawa, Tomoaki Ando, Akihisa Yoshikawa, Masahiro Nakamura, Akie Maehara, Risa Yamamoto, Yoko Okamoto, Hexing Wang, Hiromichi Yamada, Keiko Maeda, Nobuhiro Nakano, Toshiaki Shimizu, Hideoki Ogawa, Ko Okumura, Jiro Kitaura

**Affiliations:** ^1^ Department of Science of Allergy and Inflammation, Juntendo University Graduate School of Medicine, Tokyo, Japan; ^2^ Atopy (Allergy) Research Center, Juntendo University Graduate School of Medicine, Tokyo, Japan; ^3^ Department of Otorhinolaryngology, Juntendo University Graduate School of Medicine, Tokyo, Japan; ^4^ Department of Pediatrics and Adolescent Medicine, Juntendo University Graduate School of Medicine, Tokyo, Japan; ^5^ Department of Immunological Diagnosis, Juntendo University Graduate School of Medicine, Tokyo, Japan

**Keywords:** MRGPRX2, mast cell, degranulation, histamine, chymase, sensory neuron, substance P, vascular permeability

## Abstract

Mas-related G protein-coupled receptor b2 (Mrgprb2) binding to its cationic endogenous and exogenous ligands induces mast cell degranulation and promotes inflammation in mice. However, the physiological roles of its human homologue MRGPRX2 remain unclear. Here we aimed to elucidate the mechanisms by which MRGPRX2 regulates vascular permeability, and generated MRGPRX2 knock-in (MRGPRX2-KI) and Mrgprb2 knockout (Mrgprb2-KO) mice. Substance P (SP) and ciprofloxacin strongly degranulated MRGPRX2-KI peritoneal mast cells (PMCs) better than WT PMCs, whereas Dermatophagoides pteronyssinus (Der p) extract and phenol-soluble modulin α3 (PSMα3) did not degranulate PMCs. SP-stimulated MRGPRX2-KI PMCs released large amounts of histamine and mast cell protease 4 (MCPT4) chymase. Der p extract, PSMα3, and MCPT4, but not histamine, induced SP release from dorsal root ganglion (DRG) cells. However, this effect of Der p extract/PSMα3 was suppressed by a transient receptor potential vanilloid 1 (TRPV1) antagonist. SP-, ciprofloxacin-, Der p extract-, PSMα3-, and MCPT4-induced vascular permeability was highest in MRGPRX2-KI mice, which depended on SP. In addition, SP-, ciprofloxacin- and PSMα3-induced MRGPRX2-dependent vascular hyperpermeability was suppressed by antihistamine and chymase inhibitor. TRPV1 antagonist also inhibited PSMα3-induced MRGPRX2-dependent vascular hyperpermeability. Both Mrgprb2-KO and MRGPRX2-KI did not influence the histamine-induced murine vascular hyperpermeability. Overall, our results suggest that neuronal SP induces MRGPRX2-dependent mast cell degranulation, releasing histamine and chymase, which promote vascular hyperpermeability directly or indirectly via DRG cell activation. Importantly, the worsening cycle (MRGPRX2 → mast cell degranulation → chymase → DRG activation → SP → MRGPRX2) seems to play an important role in human MRGPRX2-depdendent inflammation.

## Introduction

Mast cells are tissue-resident immune cells that reside in close proximity to nerve endings and blood vessels that regulate innate and adaptive immunity via the activation of their receptors, including the high affinity immunoglobulin E (IgE) receptor (FcεRI). Crosslinking of IgE-bound FcεRI with a specific antigen (Ag) causes mast cell degranulation to immediately release chemical mediators (e.g., amines and proteases), thereby inducing allergic inflammation (1–3). In contrast, human Mas-related G protein-coupled receptor X2 (MRGPRX2) or its murine ortholog Mrgprb2 induces IgE-independent mast cell degranulation (4–8). In humans, MRGPRX2 is highly expressed in tryptase- and chymase-expressing mast cell (MC_TC_), but not in the tryptase-expressing mast cell (MC_T_) (6–8). MRGPRX2 is also expressed in different cell types, such as the small-diameter neurons of the dorsal root ganglion (DRG) (9). In mice, Mrgprb2 is exclusively expressed in the connective tissue mast cell (CTMC) resembling MC_TC_, but not in the mucosal mast cell (MMC) resembling MC_T_ (5–12). CTMCs express tryptases [e.g., mouse mast cell protease 6 (mMCPT6)] and chymases (e.g., mMCPT4), whereas MMCs express chymases (e.g., mMCPT1) (13, 14). Binding of a cationic ligand to MRGPRX2 in MC_TC_ or Mrgprb2 in CTMCs causes mast cell degranulation to release chemical mediators in a different manner than that in IgE-mediated mast cell degranulation (5–8, 15). These ligands include various cationic peptides, proteins, and chemical substances, including neuropeptides such as substance P (SP), antimicrobial peptides, major basic proteins, eosinophil peroxidase, and the US Food and Drug Administration-approved peptidergic drugs (5–8, 16–24).

Analysis of Mrgprb2 knockout (Mrgprb2-KO) mice has revealed the critical roles of Mrgprb2 in inflammatory diseases, pain, and itch (5, 25–27). Transient receptor potential vanilloid 1 (TRPV1)^+^ sensory neuron-derived SP stimulates Mrgprb2-dependent mast cell activation, causing inflammatory pain under certain conditions (25). FcεRI- or Mrgprb2-mediated mast cell activation leads to histaminergic or non-histaminergic itch, respectively, and Mrgprb2 activation releases more tryptase and less histamine from mast cells than FcεRI activation (26). Exposure to house dust mite (HDM) allergen activates TRPV1 ^+^ nociceptor to release SP, which activates mast cells via Mrgprb2, leading to the development of allergic skin inflammation (27).

Interestingly, 50% effective concentration (EC_50_) values of most ligands for Mrgprb2 are significantly higher than those for MRGPRX2 (5–8). Therefore, binding of an endogenous or exogenous ligand to MRGPRX2 may play more significant roles in the development of human inflammatory diseases. However, tools to analyze the *in vivo* functions of MRGPRX2 are currently lacking. Therefore, in this study, we established MRGPRX2 knock-in (MRGPRX2-KI) mice expressing MRGPRX2 but not Mrgprb2. We also generated Mrgprb2-KO mice, in which a fluorescent protein tdTomato was expressed under the control of the Mrgprb2 promoter.

Here, we aimed to clarify the mechanism by which MRGPRX2 regulates vascular permeability in response to external stimuli. We analyzed vascular permeability in wild-type (WT), Mrgprb2-KO, and MRGPRX2-KI mice in response to various stimuli, including known MRGPRX2/Mrgprb2 ligands. Additionally, we analyzed the degranulation and/or secretion products of mouse-derived peritoneal mast cells (PMCs) belonging to CTMCs and DRG cells in response to the same stimuli. Our results indicate that neuronal SP-driven MRGPRX2-dependent mast cell degranulation products, histamine and chymase, contribute to IgE-independent vascular hyperpermeability, implicating the sensory neuron-mast cell crosstalk in human inflammatory diseases.

## Material and methods

### Mice

In this study, all procedures were approved by the Institutional Review Committee of Juntendo University (approval numbers 2023130, 2022100, 2021189, and 2020129). C57BL/6 and BALB/c mice were purchased from Sankyo Labo Service Corporation (Tokyo, Japan). MRGPRX2-KI mice and Mrgprb2-KO mice were generated on a C57BL/6 background were generated (TransGenic Inc., Kobe, Japan). Both mice were backcrossed to a BALB/c background for eight generations. The mice (age: 6-10 weeks) were used for subsequent experiments.

### Antibodies and reagents

Ciprofloxacin and icatibant were purchased from Sigma-Aldrich (St. Louis, MO). Phenol-soluble modulin α3 (PSMα3; MEFVAKLFKFFKDLLGKFLGNN) was synthesized from GL Biochem (Shanghai, China). Dermatophagoides pteronyssinus (Der p) extract was purchased from Greer Laboratories (Lenoir, NC, USA). Enzyme-linked immuno-sorbent assay (ELISA) kits to measure leukotriene B4 (LTB4) (R&D Systems, Minneapolis, MN), cysteinyl LTs (Cayman Chemical Company, MI), histamine (MBL, Tokyo, Japan), serotonin (immuSmol, Bordeaux, France), mouse tryptase beta 2 [mouse mast cell protease 6 (mMCPT6)] (CusaBio, Houston, TX), mMCPT4 (AVIVA System Biology, Sandiego, CA), and human chymase (R&D Systems). Mouse interleukin-3 (IL-3), mouse stem cell factor (SCF), human SCF, mMCPT1, and mMCPT6 were purchased from R&D Systems. mMCPT4 was from CusaBio. 2,4-dinitrophenyl (DNP)-human serum albumin (HSA) and 2,4,6-trinitrophenyl (TNP)-bovine serum albumin (BSA) was from Sigma-Aldrich (St. Louis, MO) and Biosearch Technologies (Lystrup, Denmark), respectively. Compound 48/80 and capsaicin (Sigma-Aldrich, St Louis, MO), SP (Peptide Institute, Inc., Osaka, Japan), Cetirizine (TCI AMERICA, Portland, OR), TY-51469 (MedChemExpress, Monmouth Junction, NJ), RWJ-56110 (Tocris Bioscience, Ellisville, MO), AZ3451, I-191, AMG-517 (Selleck Chemicals LLC), AMG-9810 (Cayman Chemical, Ann Arbor, MI), DAPI (4’,6-diamidino-2-phenylindole) (FujifilmWako, Osaka, Japan), and Piperine (TCI, Tokyo, Japan) were used. Anti-SP and normal rabbit serum were purchased from Sigma-Aldrich. Anti-DNP IgE (H1-ε-26) and anti-TNP IgE (BD Biosciences, San Jose, CA) were used. All the following antibodies (Abs) were purchased from BioLegend (San Diego, CA): fluorescein isothiocyanate (FITC)-conjugated anti-mouse FcεRIα, anti-mouse CD3, anti-mouse CD4, anti-mouse CD8, anti-mouse CD11b, anti-mouse CD11c, anti-mouse CD19, anti-mouse/human B220, anti-mouse Gr-1, anti-Ly-6G, anti-mouse epithelial cell adhesion molecule (EpCAM), anti-mouse NK1.1, and anti-mouse Ter119, allophycocyanin (APC)-conjugated anti-mouse CD63, anti-human CD63, anti-human MRGPRX2, and mouse IgG2b, phycoerythrin (PE)-conjugated anti-human MRGPRX2 and mouse IgG2b, APC-cyanine 7 (Cy7)-conjugated anti-mouse CD45, PE-Cy7-conjugated anti-mouse FcεRIα, peridinin chlorophyll protein (PerCP)-Cy5.5-conjugated anti-mouse CD11b, and Brilliant Violet 421 (BV421)-conjugated-anti-mouse c-Kit.

### Cells

BMMCs and PMCs were generated as previously described (24, 28). Briefly, BM cells from mice were cultured in the Roswell Park Memorial Institute (RPMI)-1640 medium containing 10% fetal calf serum (FCS) and 10 ng/mL recombinant mouse IL-3 for five weeks to generate BMMCs. To collect the peritoneal cells, mice were intraperitoneally injected with phosphate-buffered saline (PBS) supplemented with 2% FCS. Peritoneal cells were cultured for 10 days in Iscove’s modified Dulbecco’s medium (IMDM) containing 10% FCS, 10 ng/mL recombinant mouse IL-3, and 10 ng/mL recombinant mouse SCF to collect floating cells as PMCs. DRG cells were prepared as previously described (26, 27, 29). Briefly, DRG neurons were isolated from all spinal levels in mice, and incubated with Hanks’ balanced salt solution (HBSS) (Thermo Fisher Scientific, Waltham, MA) supplemented with 5 ng/mL dispase II (FujifilmWako), and 1 mg/mL collagenase Type I (Worthington, Lakewood, NJ) at 37°C for 20 min. After mechanically agitation, the DRG cells were filtered through cell strainers. The dissociated cells were spun at 300g for 5 min, resuspended with Dulbeccos Modification of Eagles Medium (DMEM)/Ham’s F-12 (FujifilmWako), plus 10% fetal bovine serum (FBS), and cultured on 96-well plates coated with poly-D-lysine and laminin at 37°C for 2 h. After discarding the supernatant, DRG cells were cultured in Neurobasal Plus medium (Thermo Fisher Scientific) supplemented with GlutaMAX-I (100x) (Gibco), CultureOne Supplement (100x) (Gibco), B-27 Plus Supplement (50x) (Gibco),50 ng/mL glial cell-line derived neurotrophic factor (GDNF) (BioLegend), and 50 ng/mL nerve growth factor (NGF) (BioLegend) at 37°C for 7 days before experiments. The human mast cell line LAD2 was maintained in StemPro-34 serum-free media (SFM) (Life Technologies) in the presence of 100 ng/mL recombinant human SCF (24).

### Evaluation of vascular permeability in mice

Mice were intradermally injected with the indicated amounts of 20 or 100 ng of compound 48/80, 5 or 40 μg ciprofloxacin, 1.75 μg icatibant, 3, 8, or 25 pmol SP, 1 or 10 μg Der p extract, 0.2 or 2 μg PSMα3, 200 ng MCPT4, and 100 μg histamine or PBS in each ear just before intravenous injection with 0.5% Evans blue dye (Sigma, St Louis, MO). Alternatively, mice were intradermally injected with 50 ng anti-DNP IgE (H1-ϵ-26) in each ear 24 h before intravenously injection with 0.5% Evans blue dye containing 250 μg DNP-HSA. In any case, 30 min after an intravenous injection of dye, the removed and finely cut ear tissues were incubated in 0.5 mL of 1N KOH overnight at 37°C with shaking. Then, 0.25 mL of 1N phosphoric acid and 0.65 mL of acetone were added to the collected supernatant. After centrifugation at 700 g for 15 min, 0.3 mL of the supernatant was added to a 96-well microplate. To evaluate the extravasated dye amount, the absorbance was measured at 620 nm using a 96-well microplate luminometer, as previously described (24, 28, 30). In some experiments, 15 μl of anti-SP serum or control serum together with 40 μg ciprofloxacin, 10 μg Der p extract, 2 μg PSMα3, 200 ng mMCPT4, 100 μg histamine, or 50 ng anti-DNP IgE was injected intradermally into each ear of the mice. Six hundred μg cetirizine or 50 μg TY-51469 was intraperitoneally injected 0.5 or 2 h before an intradermal injection of 25 pmol SP, 40 μg ciprofloxacin, 2 μg PSMα3, 250 μg TNP-HSA, or vehicle in each ear of the mice. Alternatively, WT, Mrgprb2-KO, and MRGPRX2-KI mice were orally administered with 20 μg of AMG517 or vehicle. After 7 min, these mice were intradermally injected with 2 μg PSMα3 in each ear immediately before intravenous injection of 0.5% Evans blue dye. After 10 min, the amount of extravasated dye was measured as described above.

### Degranulation assay in mast cells

BMMC, PMC, or LAD2 cells were stimulated with the indicated concentrations of compound 48/80, SP, Der p extract, or PSMα3, 10 μg/mL ciprofloxacin, or 10 μM icatibant, for 30 min in Tyrode’s buffer (112 mM NaCl, 2.7 mM KCl, 0.4 mM NaH_2_PO_4_, 1.6 mM CaCl_2_, 1.0 mM MgCl_2_, 5.6 mM glucose, 10 mM HEPES, and 0.1% Gelatin). Alternatively, PMC or LAD2 cells were sensitized with 0.5 μg/mL or 1 μg/mL anti-TNP IgE overnight, respectively, and then stimulated with indicated concentrations of TNP-BSA or SP for 30 min in Tyrode’s buffer. The magnitude of degranulation was assessed by measuring percentages of β-hexosaminidase release or surface CD63-positice mast cells (24, 28). β-hexosaminidase release was calculated by dividing fluorescence in supernatant by fluorescence in cell lysate. After stimulation, cell supernatants or lysates were incubated in citrate buffer (25 mM citric acid, 25 mM Trisodium citrate, pH 4.5) with 0.3 mg/mL p-nitrophenyl N-acetyl-β-d-glucosaminide (Sigma-Aldrich) for 60 min at 37°C. Cell lysates were prepared by lysing cell pellets with 1% Triton X-100. The reactions were terminated by adding 50 mM sodium carbonate buffer. OD at 405 nm was determined using an ELISA plate reader. Alternatively, the percentage of surface CD63-positive mast cells, corresponding to degranulated mast cells, within the total number of mast cells was measured using flow cytometry.

### Measurement of chemical mediators released from DRG and mast cells

DRG cells were incubated in the Neurobasal Plus medium with the indicated concentrations of capsaicin, Der p extract, and PSMα3, 5 μg/mL of mMCPT1, mMCPT4, or mMCPT6, 1 μM histamine, or vehicle for 1 h after pre-incubation in the presence of 10 μM TY-51469, 10 μM RWJ-56110, 10 μM AZ3451, 1 μM AMG517, and vehicle for 1 h. Levels of SP in the supernatant of DRG cells were measured via enzyme-linked immuno-sorbent assay (ELISA). Levels of histamine or human chymase in the culture supernatants of LAD2 cells and levels of histamine, serotonin, mouse tryptase beta 2 (mMCPT6), and mMCPT4 in the culture supernatants of MRGPRX2-KI PMCs were measured using ELISA. LAD2 cells and MRGPRX2-KI PMCs were sensitized with 1 or 0.5 μg/mL anti-TNP IgE overnight, respectively, and stimulated with the indicated concentrations of TNP-BSA and SP for 30 min in Tyrode’s buffer. Levels of LTB_4_ and cysteinyl LTs in the culture supernatants of WT, Mrgprb2-KO, and MRGPRX2-KI PMCs stimulated with the indicated concentrations of SP for 1 h were measured using ELISA.

### Real-time polymerase chain reaction

Total RNA was extracted from the PMCs and DRG cells using the RNeasy Mini Kit (Qiagen), according to the manufacturer’s instructions. cDNA was synthesized from the total RNA using the ReverTra Ace qPCR RT kit (Toyobo). Real-time PCR was performed using the Step One Plus Real-Time PCR System (Thermo Fisher Scientific) with the SYBR Green PCR Master Mix (Applied Biosystems, Life Technologies) (31). The following primers were used: 5′-GGAACCAAGCCATGATTTTGC-3′ (forward) and 5′- GTGAAGGCATTCGTGTGCATA-3′ (reverse) for *mrgprb2*; 5′- CACAGACCAGTTTAACACTTCC-3′ (forward) and 5′- CTCTTTGATGACCTCCTCGC-3′ (reverse) for *tdTomato*; 5′- CACAGACCAGTTTAACACTTCC-3′ (forward) and 5′- GATCAGGGTCTCCTTGCCAC-3′ (reverse) for knock-in *MRGPRX2;* and 5′- GCAGAAGAAGGGCTTGGTCA -3′ (forward) and 5′-CCGGAATCGAACCCTGATT-3′ (reverse) for mouse *18S rRNA.* The mRNA expression levels were quantified using the comparative method with the StepOne Software, and housekeeping gene 18S rRNA levels were used for normalization.

### Flow cytometry

Flow cytometric analysis was performed using FACSVerse (BD Biosciences) equipped with FlowJo software (Tree Star). BMMCs, PMCs, peritoneal lavage cells, and skin cells were used in this study. To isolate the ear skin cells (31), skin samples were minced with scissors and incubated with the RPMI-1640 medium with 2 mg/mL collagenase type I (FUJIFILM) and 0.1 mg/mL DNase I (Roche) for 1 h at 37°C. Then, the cell suspension was incubated with 10 mM ethylene diamine tetra acetic acid (EDTA) for 5 min at 37°C. After washing, the cells were resuspended in a buffer for flow cytometry. Mast cells in the small intestine and skin were identified as CD3^-^CD4^-^CD8^-^CD11b^-^CD11c^-^CD19^-^Gr-1^-^EpCAM^-^NK1.1^-^Ter119^-^CD45^+^c-Kit^+^FcεRIα^+^ cells. Mast cells in the peritoneal cavity were identified as CD45^+^c-Kit^+^FcεRIα^+^ cells. BMMCs and PMCs were identified as c-Kit^+^FcεRIα^+^ cells. Skin neutrophils were identified as CD45^+^Ly-6G^+^CD11b^+^ cells. Degranulated mast cells were identified as CD63^+^ cells within mast cells.

### Histological analysis

Histological analyses were performed as previously described (30–32). Sections of the ear and back skin were stained with toluidine blue (pH 4.1) to calculate the total mast cell number and percentage of degranulated mast cells among the total mast cells in the ear and back skin.

### Statistical analyses

**Figure 1:**
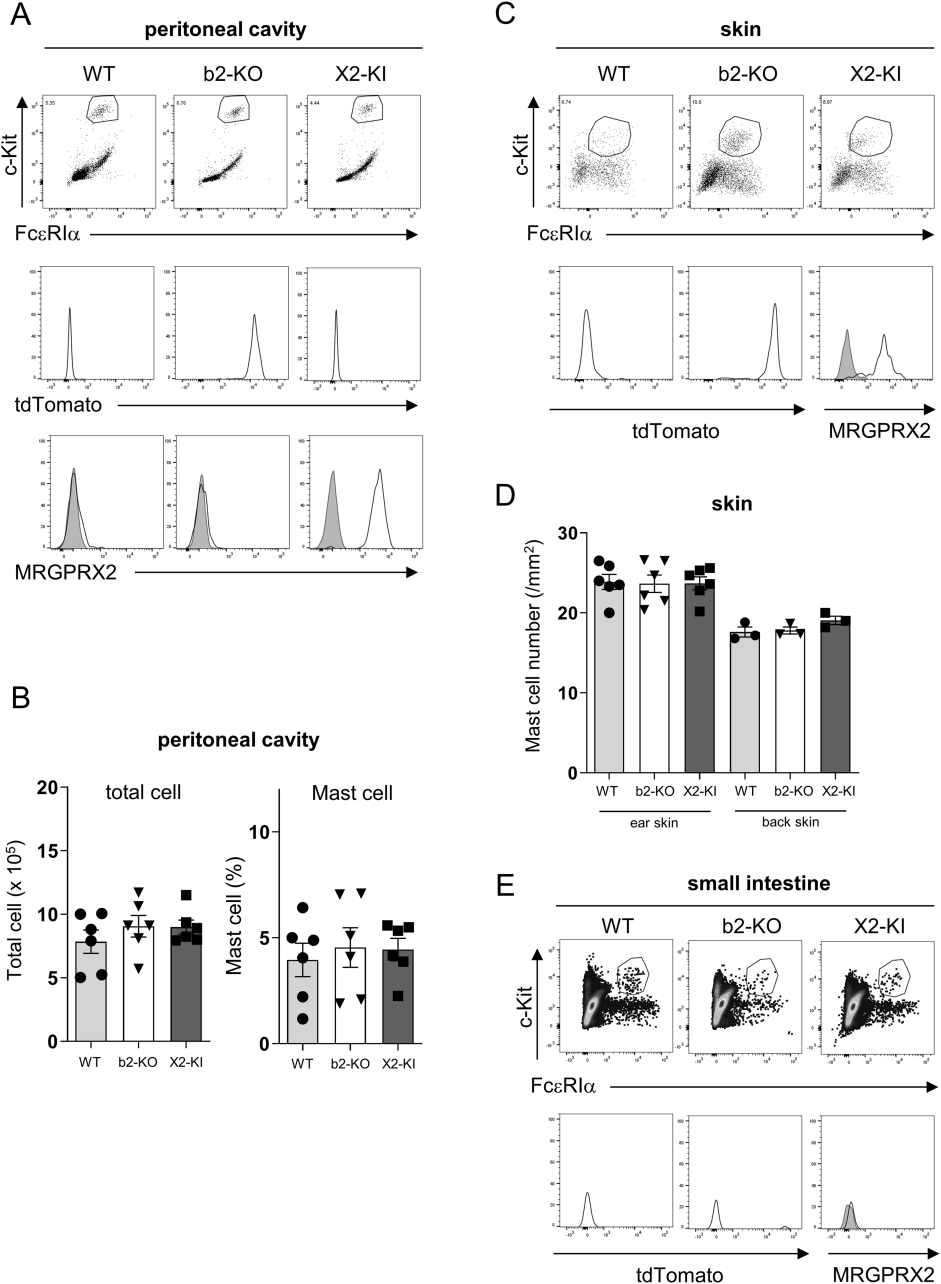
Generation of MRGPRX2-KI mice. **(A, C, E)** Surface expression levels of FcεRIα and c-Kit (upper panel) in the peritoneal **(A)**, skin **(C)**, and small intestinal **(E)** cells. Expression levels of tdTomato (middle panel) and surface expression levels of MRGPRX2 (lower panel) in FcεRIα^+^c-Kit^+^ peritoneal mast cells **(A)**, skin mast cells **(C)**, and small intestinal mast cells **(E)** in the WT, Mrgprb2-KO (b2-KO), and MRGPRX2-KI (X2-KI) mice. Data are representative of three independent experiments. Control staining is shown in the shaded histograms. **(B, D)** Total cell numbers and percentages of FcεRIα^+^c-Kit^+^ mast cells in the peritoneal cavity **(B)** and numbers of toluidine blue-positive mast cells in the ear or back skin **(D)** of WT, Mrgprb2-KO, and MRGPRX2-KI mice. n = 3-6; ± SD.

**Figure 2:**
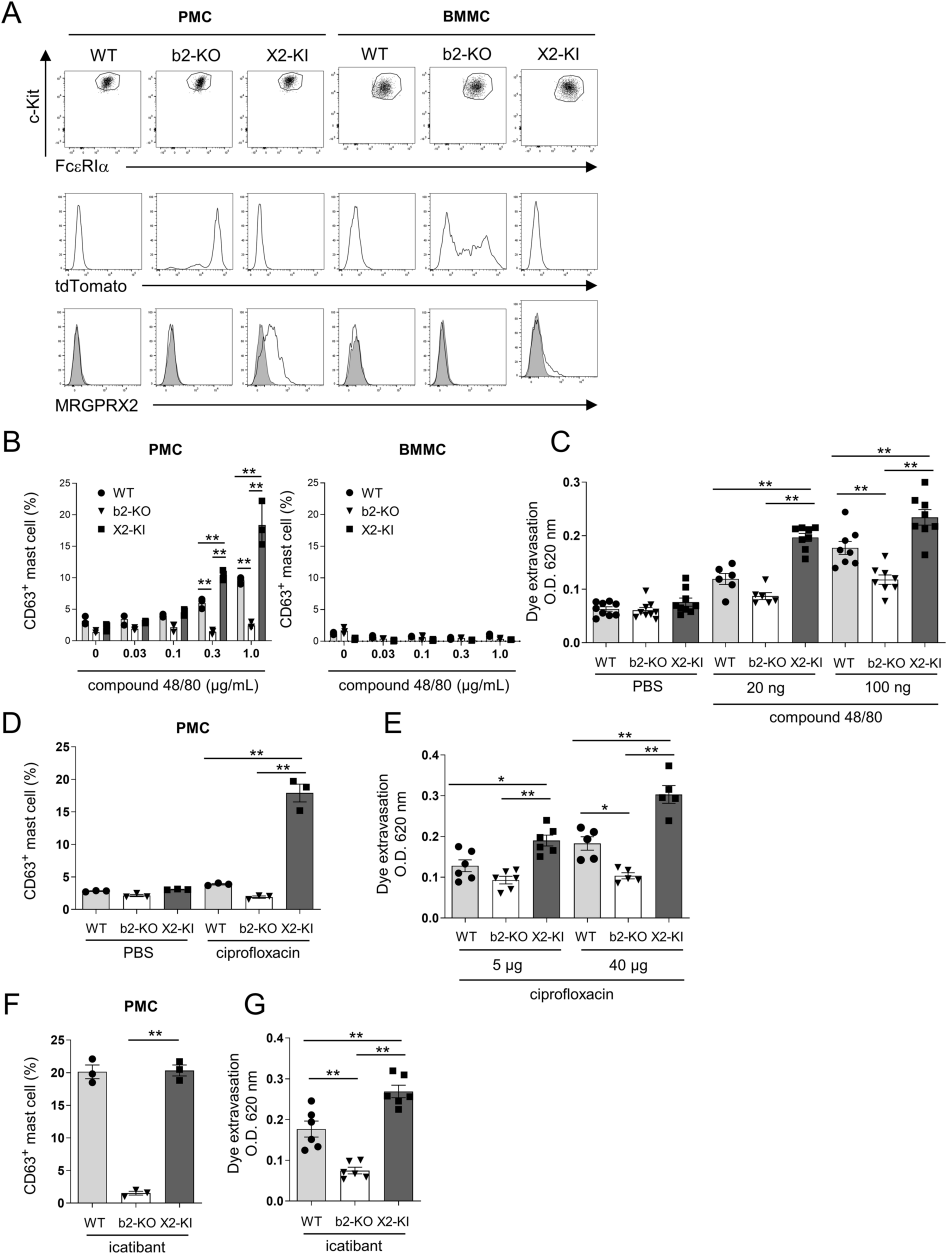
Binding of compound 48/80 or ciprofloxacin to MRGPRX2 strongly promotes PMC degranulation and murine vascular permeability more than the binding to Mrgprb2. **(A)** Surface expression levels of FcεRIα and c-Kit (upper panel) and MRGPRX2 (middle panel) and expression levels of tdTomato (lower panel) in the PMCs from WT, Mrgprb2-KO (b2-KO), and MRGPRX2-KI (X2-KI) mice. Control staining is shown in the shaded histograms. **(B, D, F)** Percentages of surface CD63-positive cells in WT, Mrgprb2-KO, and MRGPRX2-KI PMCs and BMMCs **(B)** and PMCs **(D, F)** after treatment with the indicated concentrations of compound 48/80 **(B)**, 10 μg/mL ciprofloxacin **(D)**, or 10 μg/mL icatibant **(F)**. Data are representative of three independent experiments and indicate the mean ± SD. *P < 0.05 and **P < 0.01. **(C, E, G)** Quantification of the Evans blue dye that extravasated into the ear skin in WT, Mrgprb2-KO, and MRGPRX2-KI mice after treatment with indicated amounts of compound 48/80 **(C)**, ciprofloxacin **(E)**, and 1.75 μg icatibant **(G)** or phosphate-buffered saline (PBS). n = 5-9; ± SD. *P < 0.05 and **P < 0.01.

**Figure 3:**
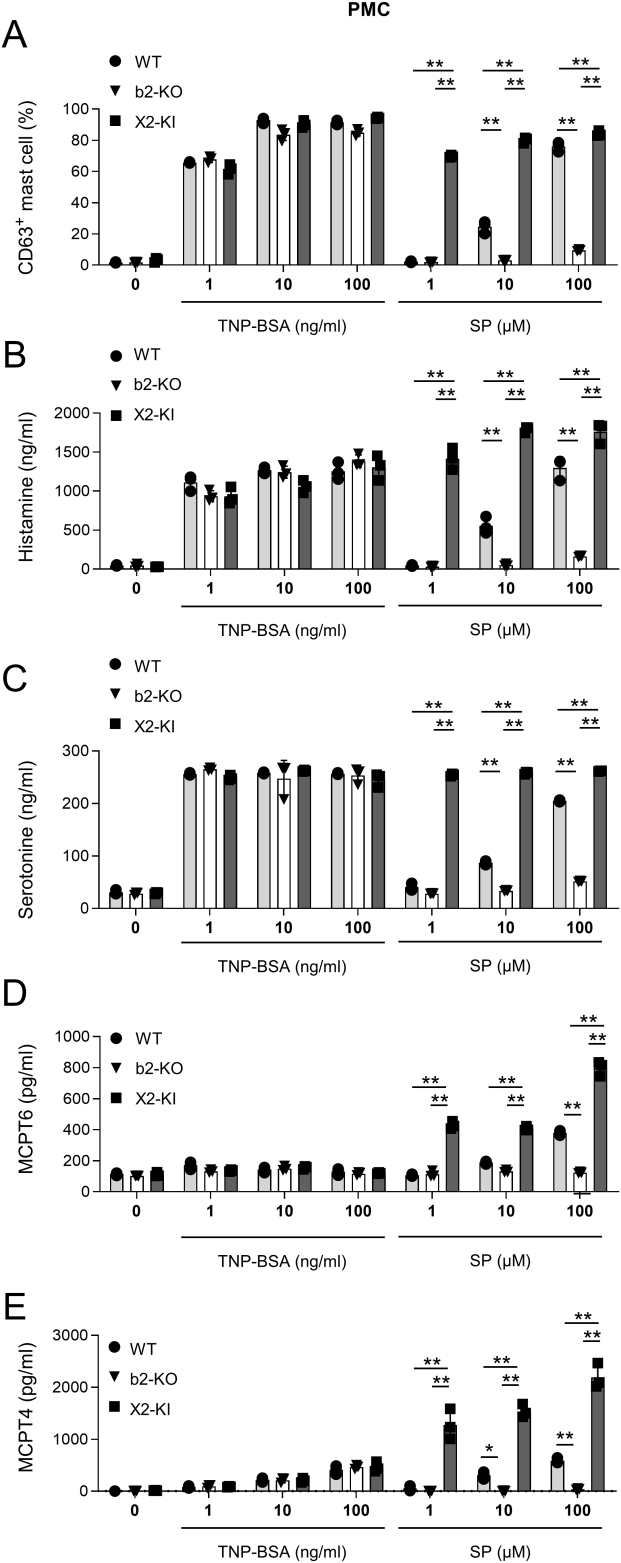
SP-stimulated MRGPRX2-KI PMCs released larger amounts of amines and proteases than the WT counterparts. **(A–E)** Percentages of surface CD63^+^ PMCs **(A)** and levels of histamine **(B)**, serotonin **(C)**, mMCPT6 **(D)**, and mMCPT4 **(E)** in the culture supernatants of anti-TNP IgE-sensitized WT, Mrgprb2-KO (b2-KO), and MRGPRX2-KI (X2-KI) PMCs after treatment with the indicated concentrations of TNP-BSA and SP. Data are representative of three independent experiments. *P < 0.05 and **P < 0.01.

**Figure 4:**
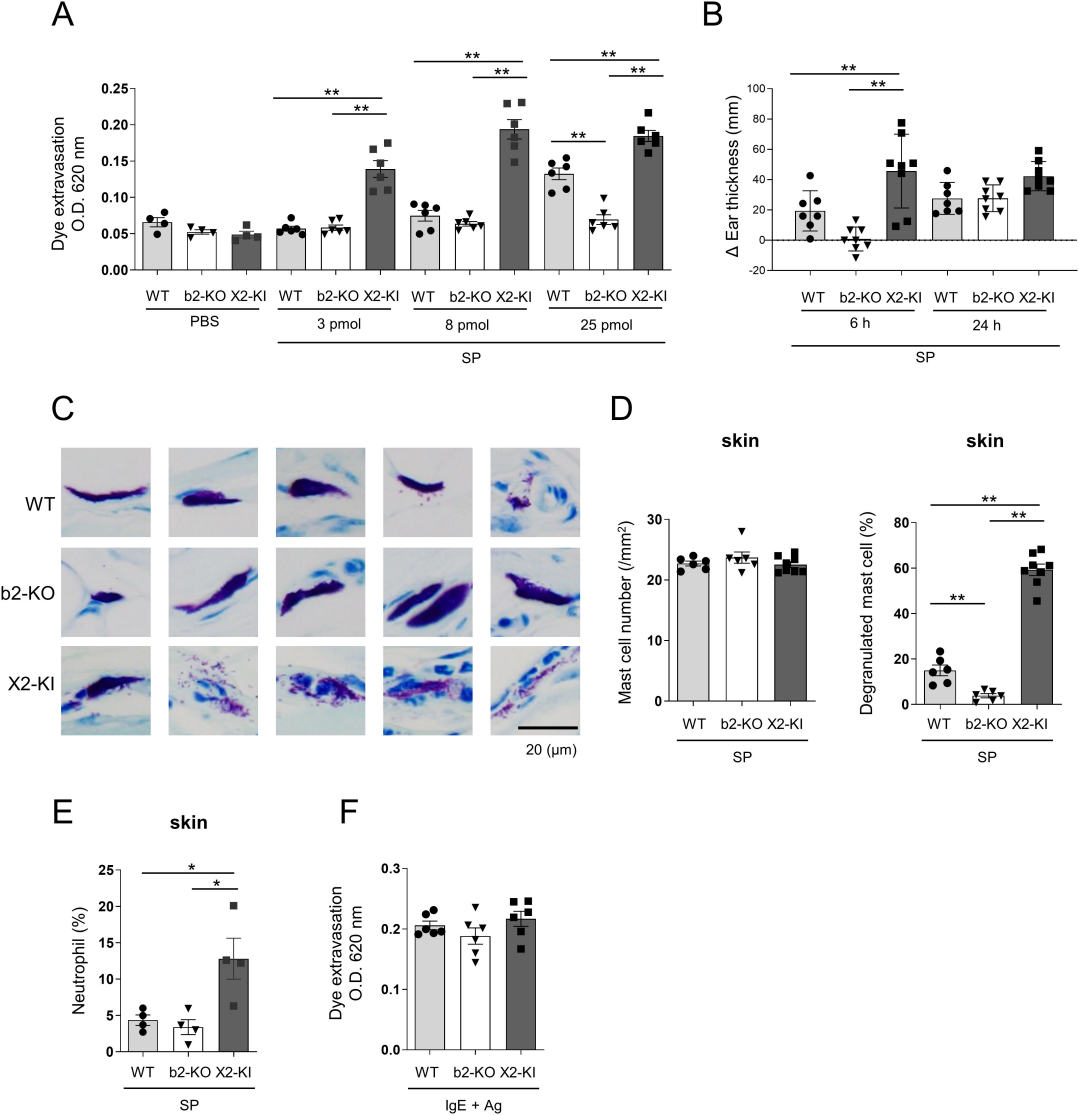
Intradermal injection of SP induces higher vascular permeability in MRGPRX2-KI mice compared to that in WT mice. **(A, F)** Quantification of the Evans blue dye that extravasated into the ear skin in WT, Mrgprb2-KO (b2-KO), and MRGPRX2-KI (X2-KI) mice after treatment with indicated amounts of SP **(A)** and anti-DNP IgE plus DNP-HSA **(F)**. **(B-E)** WT, Mrgprb2-KO, and MRGPRX2-KI mice 6 and 24 h **(B)** or 6 h **(D, E)** after the intradermal injection of 25 pmol SP. **(B)** Ear thickness. **(C)** Representative images of toluidine blue (pH 4.1)-stained mast cells in the ear skin sections (Scale bar; 20 μm). **(D)** Total number of mast cells (left panel) and percentage of degranulated mast cells (right panel) in the ear skin. **(E)** Percentage of neutrophils. **(A, B, D-F)** n = 4-8; ± SD. *P < 0.05 and **P < 0.01.

**Figure 5:**
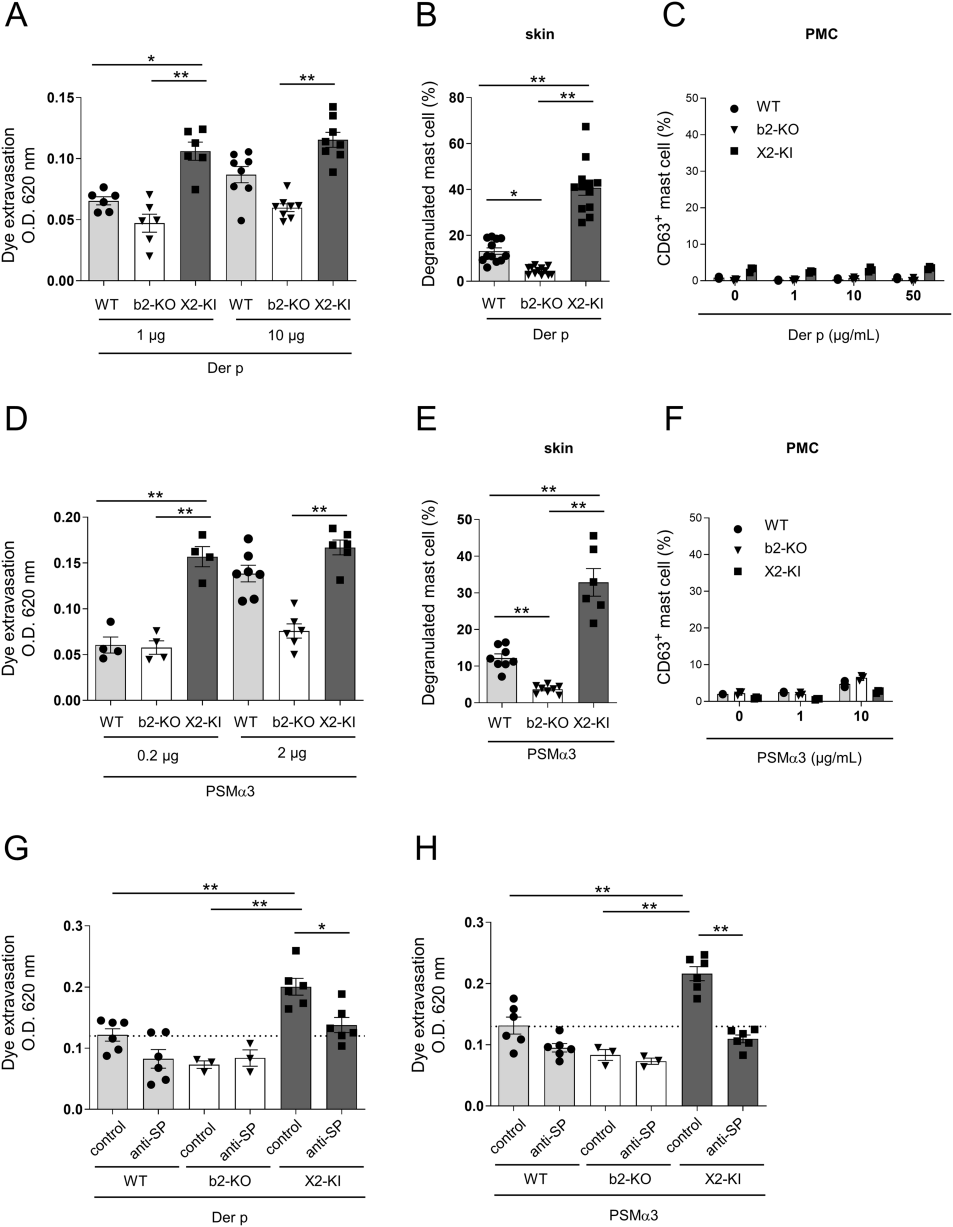
Stimulation with the Der P extract and PSMα3 increases vascular permeability via SP-driven MRGPRX2 signal in the skin mast cells. **(A, D)** Quantification of the Evans blue dye that extravasated into the ear skin in the WT, Mrgprb2-KO (b2-KO), and MRGPRX2-KI (X2-KI) mice intradermally injected with the indicated amounts of the Der p extract **(A)** and PSMα3 **(D)**. **(B, E)** Percentages of degranulated skin mast cells in the WT, Mrgprb2-KO and MRGPRX2-KI 6 h after the intradermal injection of 10 μg Der p extract **(B)** and 2 μg PSMα3 **(E)**. **(C, F)** Percentages of surface CD63^+^ PMCs from the WT, Mrgprb2-KO, and MRGPRX2-KI mice after stimulation with the indicated concentrations of Der p extract **(C)** and PSMα3 **(F)**. **(G, H)** Quantification of the Evans blue dye that extravasated into the ear skin in the WT, Mrgprb2-KO, and MRGPRX2-KI mice intradermally injected with 10 μg Der p extract **(G)** and 2 μg PSMα3 **(H)** along with the anti-SP or control serum. **(A, B, D, E, G, H)** n = 3-10; ± SD. *P < 0.05 and **P < 0.01. **(C, F)** Data are representative of three independent experiments.

**Figure 6:**
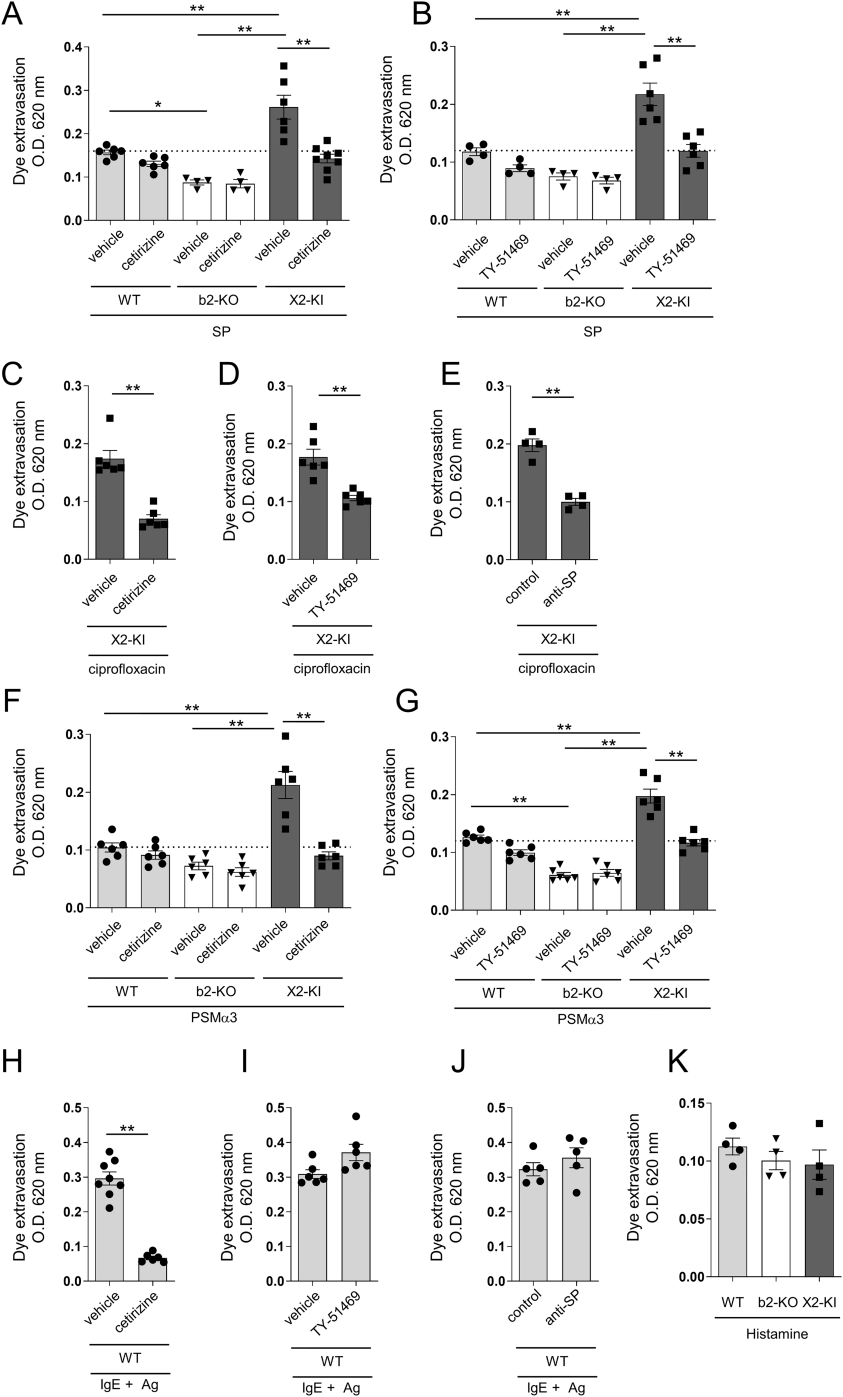
SP-, ciprofloxacin- or PSMα3-stimulated MRGPRX2-dependent vascular hyperpermeability is suppressed by antihistamine or chymase inhibitor. **(A-K)** Quantification of the Evans blue dye that extravasated into the ear skin. **(A, B, F, G)** WT, Mrgprb2-KO (b2-KO), and MRGPRX2-KI (X2-KI) mice were intradermally injected with 25 pmol SP **(A, B)** and 2 μg PSMα3 **(F, G)**. **(C-E, H-J)** MRGPRX2-KI mice were intradermally injected with 40 μg ciprofloxacin **(C-E)**. WT mice were intradermally injected with anti-DNP IgE, followed by intravenous injection of DNP-HSA **(H-J)**. Effects of 600 μg cetirizine or vehicle **(A, C, F, H)**, 50 μg TY-51469 or vehicle **(B, D, G, I)**, and 15 μL of anti-SP or control serum **(E, J)** on vascular permeability. **(K)** WT, Mrgprb2-KO, and MRGPRX2-KI mice were intradermally injected with 100 μg histamine. n = 4-10; ± SD. *P < 0.05 and **P < 0.01. Data are representative of two independent experiments.

**Figure 7:**
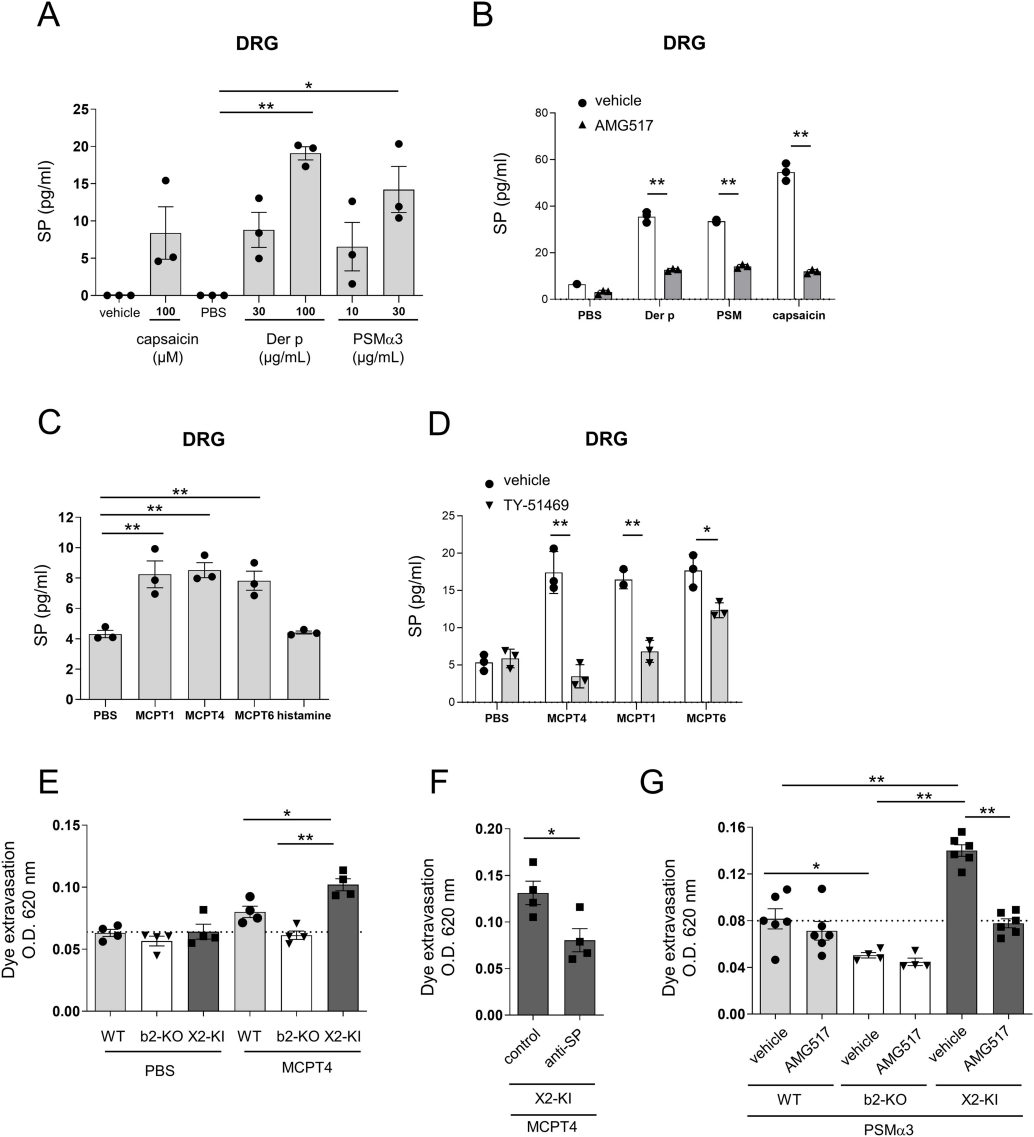
Der p extract, PSMα3, and chymase, but not histamine, stimulated the release of SP from DRG cells. **(A-D)** Levels of SP in the culture supernatants of DRG cells stimulated with the indicated concentrations of Der p extract, PSMα3, capsaicin, and vehicle **(A)**, 30 μg/mL Der p extract, 30 μg/mL PSMα3, 100 μM capsaicin, and PBS in the presence of AMG517 or vehicle **(B)**, 5 μg/mL of mMCPT1, mMCPT4, and mMCPT6, 1 μM histamine, and vehicle **(C)**, and 5 μg/mL of mMCPT1, mMCPT4, MCPT6, and PBS in the presence of TY-51469 or vehicle **(D)**. *P < 0.05 and **P < 0.01. Data are representative of three independent experiments. **(E-G)** Quantification of the Evans blue dye that extravasated into the ear skin in the WT, Mrgprb2-KO (b2-KO), and MRGPRX2-KI (X2-KI) mice intradermally injected with 200 ng mMCPT4 or vehicle **(E)**, MRGPRX2-KI mice intradermally injected with 200 ng mMCPT4 along with anti-SP or control serum **(F)**, or WT, Mrgprb2-KO, and MRGPRX2-KI mice intradermally injected with 2 μg PSMα3 before oral administration of AMG517 or vehicle **(G)**. n = 4-6; ± SD. *P < 0.05 and **P < 0.01. Data are representative of two independent experiments.

Statistical analysis was performed using Prism 8 software (GraphPad). Data are expressed as the means ± standard deviation (SD). Ordinary one-way analysis of variance (ANOVA) with Tukey’s multiple comparisons was used in **Figures 1**–**5**, **6A, B, F, G**, **7**; **Supplementary Figures S2**, **S4**–**S6**, **S8**. Unpaired two-tailed Student’s t test with Welch’s correction was used in **Figures 6C-E, H-J**; **Supplementary Figure S7**. The differences were compared between two groups or among multiple groups. *p < 0.05 and **p < 0.01 were considered to be statistically significant.

## Results

### Generation of MRGPRX2-KI mice

To clarify the physiological roles of MRGPRX2 in mast cells, we established MRGPRX2-KI mice with a C57BL/6 background (**Supplementary Figure S1A**). Consistent with previous studies (5–8), flow cytometric analysis using an anti-MRGPRX2 antibody (Ab) revealed that surface MRGPRX2 was expressed in FcεRIα^+^c-Kit^+^ mast cells in the peritoneal cavity of MRGPRX2-KI mice (**Figure 1A**). In the process of generating MRGPRX2-KI mice, we also established Mrgprb2-KO mice, expressing the fluorescent protein tdTomato under the control of the Mrgprb2 promoter. Mast cells in the peritoneal cavity of Mrgprb2-KO mice did not express surface MRGPRX2 but expressed tdTomato (**Figure 1A**). Neither Mrgprb2-KO nor MRGPRX2-KI influenced the mast cell numbers in the peritoneal cavity (**Figure 1B**). Skin mast cells form MRGPRX2-KI and Mrgprb2-KO mice expressed surface MRGPRX2 and tdTomato, respectively (**Figure 1C**). Staining of skin tissue sections showed that WT, Mrgprb2-KO, and MRGPRX2-KI mice had equivalent numbers of mast cells in the ear and back skin (**Figure 1D**). In contrast, mast cells in the small intestine had no detectable levels of tdTomato or surface MRGPRX2 in all mice groups (**Figure 1E**). MRGPRX2-KI and Mrgprb2-KO mice exhibited surface MRGPRX2 and tdTomato expression, respectively, in CTMCs.

### Binding of compound 48/80 or ciprofloxacin to MRGPRX2 strongly promotes PMC degranulation and murine vascular permeability more than the binding to Mrgprb2

Next, we generated PMCs and bone marrow-derived mast cells (BMMCs) from WT, Mrgprb2-KO, and MRGPRX2-KI mice, exhibiting similar levels of FcεRIα and c-Kit for each mast cell type. MRGPRX2 was consistently expressed on the surfaces of MRGPRX2-KI PMCs. High levels of tdTomato were detected in MRGPRX2-KO PMCs. Expression of tdTomato was also observed in Mrgprb2-KO BMMCs, but its levels were lower than those in Mrgprb2-KO PMCs. However, surface expression of MRGPRX2 was undetectable in MRGPRX2-KI BMMCs (**Figure 2A**). Real-time polymerase chain reaction (PCR) analysis confirmed that *Mrgprb2*, *tdTomoato*, and *MRGPRX2* were highly expressed at the mRNA level in WT, Mrgprb2-KO, and MRGPRX2-KI PMCs, respectively. Additionally, *Mrgprb2* mRNA was not detected in WT DRG cells (**Supplementary Figure S1B**). Then, we measured the degranulation rate in PMCs and BMMCs stimulated with compound 48/80, a known Mrgprb2/MRGPRX2 ligand. Stimulation with compound 48/80 dose-dependently increased the percentages of degranulation in WT and MRGPRX2-KI PMCs; however, MRGPRX2-KI PMCs were more strongly degranulated than WT PMCs (**Figure 2B**). Mrgprb2-KO PMCs and WT, Mrgprb2-KO, and MRGPRX2-KI BMMCs did not significantly degranulate in response to compound 48/80 (**Figure 2B**; **Supplementary Figure S2**). Next, WT, Mrgprb2-KO, and MRGPRX2-KI mice were intradermally injected in ears with compound 48/80 or PBS (24). Measuring the amount of the extravasated dye showed that in response to 100 ng compound 48/80, all mice exhibited significant levels of dye extravasation in the following order: MRGPRX2-KI > WT > Mrgprb2-KO mice (**Figure 2C**). However, dye extravasation was only evident in MRGPRX2-KI mice in response to 20 ng of compound 48/80 (**Figure 2C**). When PMCs were stimulated with the fluoroquinolone antibiotic ciprofloxacin, a different Mrgprb2/MRGPRX2 ligand, remarkable degranulation was observed only in MRGPRX2-KI PMCs under the conditions tested (**Figure 2D**). Consistently, intradermal injection of a low amount (5 μg) of ciprofloxacin increased vascular permeability only in MRGPRX2-KI mice (**Figure 2E**). However, stimulation with the bradykinin B2 receptor antagonist icatibant, another Mrgprb2/MRGPRX2 ligand, caused comparable levels of degranulation in WT and MRGPRX2-KI PMCs under the conditions tested (**Figure 2F**). Icatibant-induced vascular permeability was slightly higher in the MRGPRX2-KI mice than in the WT mice (**Figure 2G**). Neither ciprofloxacin nor icatibant stimulation elevated the degranulation rate of Mrgprb2-KO PMCs or vascular permeability in Mrgprb2-KO mice.

### SP-stimulated MRGPRX2-KI PMCs release larger amounts of amines and proteases than WT mice

We generated PMCs from WT, Mrgprb2-KO, or MRGPRX2-KI mice with a BALB/c background, and confirmed that surface MRGPRX2 or tdTomato was expressed in MRGPRX2-KI or Mrgprb2-KO PMCs, respectively (**Supplementary Figure S3**). We then measured the degranulation rate and the amounts of the degranulation products histamine, serotonin, tryptase MCPT6, and chymase MCPT4 in PMCs stimulated by FcεRI engagement or SP (5–8). Mrgprb2-KO or MRGPRX2-KI did not significantly influence FcεRI-activated PMC degranulation and its products (**Figures 3A-E**). However, stimulation with SP resulted in stronger degranulation of MRGPRX2-KI PMCs than WT PMCs (**Figure 3A**). Consistently, stimulation with SP induced the release of higher amounts of all amines and proteases tested, from MRGPRX2-KI PMCs than from WT PMCs. In contrast to a recent report on Mrgprb2 versus FcεRI (26), SP-stimulated MRGPRX2-dependent degranulation evoked robust histamine and serotonin secretion in PMCs. Nonetheless, SP-stimulated MRGPRX2 activation, but not FcεRI activation, induced high levels of MCPT6 and MCPT4 release from PMCs (**Figures 3B-E**). When IgE-sensitized human mast cell line LAD2 cells were stimulated with a specific Ag or SP, we found that stimulation with SP induced degranulation to release higher levels of histamine and chymase in LAD2 cells than stimulation with FcεRI engagement (**Supplementary Figure S4**). Additionally, SP induced the release of higher amounts of LTB4 and cysteinyl LT in MRGPRX2-KI PMCs than in WT PMCs (**Supplementary Figure S5**). We found that pretreatment with Piperine, a known MRGPRX2 antagonist, suppressed SP- or compound 48/80-induced degranulation in MRGPRX2-KI PMCs (33) (**Supplementary Figure S6**), confirming that SP activated MRGPRX2-KI PMCs via MRGPRX2. Therefore, SP-stimulated MRGPRX2 activation induces the release of robust amounts of amines and proteases in mast cells.

### Intradermal injection of SP induces high vascular permeability in MRGPRX2-KI mice compared to that in WT mice

To further examine the *in vivo* effect of SP on vascular permeability, WT, Mrgprb2-KO, and MRGPRX2-KI mice were intradermally injected with different amounts of SP. Significantly increased vascular permeability was observed in WT and MRGPRX2-KI mice in response to high amounts of SP (25 pmol/ear), although the latter exhibited higher vascular permeability than the former (**Figure 4A**). Consistent with this, we observed marked ear thickness in MRGPRX2-KI mice compared to Mrgprb2-KO mice 6 h after treatment with SP (**Figure 4B**). Staining of ear tissue sections revealed that the percentage of degranulated mast cells was higher in MRGPRX2-KI mice than in WT mice. No significant differences in mast cell numbers in the ear skin were observed among the three types of mice (**Figures 4C, D**). Additionally, neutrophil infiltration in the ear skin was evident in MRGPRX2-KI mice 6h after injection of SP (**Figure 4E**), Notably, a significant increase in the vascular permeability was observed only in MRGPRX2-KI mice in response to low amounts of SP (3 or 8 pmol/ear) (**Figure 4A**). In contrast, Mrgprb2-KO or MRGPRX2-KI did not affect vascular permeability induced by FcεRI engagement (**Figure 4F**). Therefore, intradermal injection of SP strongly induced vascular hyperpermeability via enhanced degranulation of skin mast cells in MRGPRX2-KI mice.

### Intradermal injections of Der P extract and PSMα3 increase vascular permeability via SP-driven MRGPRX2-dependent mast cell degranulation

As HDM allergens and *Staphylococcus aureus* toxins regulate skin inflammation in various conditions, including atopic dermatitis (27, 34–37), we tested whether MRGPRX2 regulates vascular permeability induced by HDM Dermatophagoides pteronyssinus (Der p) extract or *S. aureus*-secreting toxin phenol-soluble modulin α3 (PSMα3). Intradermal injection of the Der p extract significantly increased the amount of extravasated dye in both WT and MRGPRX2-KI mice; however, the latter exhibited extravasation of higher dye amounts than the former (**Figure 5A**). Additionally, we observed more degranulated skin mast cells in MRGPRX2-KI mice than in WT mice (**Figure 5B**). However, stimulation with Der p extract failed to induce PMC degranulation, irrespective of Mrgprb2-KO or MRGPRX2-KI (**Figure 5C**). No significant increase in vascular permeability or degranulation of skin mast cells was observed in Mrgprb2-KO mice (**Figures 5A, B**). Similarly, intradermal injection of PSMα3 caused extravasation of higher amounts of dye and higher percentages of degranulated skin mast cells in MRGPRX2-KI mice than in WT mice (**Figures 5D, E**). Stimulation with PSMα3 caused no significant PMC degranulation (**Figure 5F**). Interestingly, treatment with anti-SP serum decreased Der p extract- or PSMα3-induced vascular hyperpermeability in MRGPRX2-KI mice to levels comparable to those in control serum-treated WT counterparts (**Figures 5G, H**). These results indicated that intradermal injection of Der p extract or PSMα3 increases vascular permeability via SP-driven MRGPRX2-dependent mast cell degranulation.

### SP-, ciprofloxacin- and PSMα3-stimulated MRGPRX2-dependent vascular hyperpermeability is suppressed by treatment with antihistamine or chymase inhibitor

Because MRGPRX2-dependent PMC degranulation strongly induced the release of histamine and chymase, we investigated whether histamine and/or chymase contributed to vascular hyperpermeability in MRGPRX2-KI mice. The results showed that the amount of extravasated dye in SP-injected MRGPRX2-KI mice was reduced by treatment with the antihistamine cetirizine or the chymase inhibitor TY-51469, to the levels of SP-injected, vehicle-treated WT mice, indicating that SP-induced vascular hyperpermeability in MRGPRX2-KI mice depended on histamine and chymase (**Figures 6A, B**). Additionally, ciprofloxacin-induced vascular hyperpermeability in MRGPRX2-KI mice was inhibited by cetirizine, TY-51469, and anti-SP serum (**Figures 6C-E**). Moreover, PSMα3-induced vascular hyperpermeability in MRGPRX2-KI mice was lowered by treatment with cetirizine or TY-51469, to the levels observed in PSMα3-induced, vehicle-treated WT mice (**Figures 6F, G**). However, IgE/Ag-induced vascular hyperpermeability in WT mice was abrogated by treatment with cetirizine, but not with TY-51469 or anti-SP serum (**Figures 6H, J**). We confirmed that Mrgprb2-KO or MRGPRX2-KI did not affect histamine-induced vascular hyperpermeability in mice (**Figure 6K**) and that histamine-stimulated vascular hyperpermeability in WT mice was not affected by treatment with anti-SP serum (**Supplementary Figure S7**). Therefore, MRGPRX2-dependent mast cell degranulation products, histamine and chymase, strongly promote vascular permeability.

### Der p extract, PSMα3, and chymase, but not histamine, stimulate the release of SP from DRG cells

To examine the involvement of neuronal SP in MRGPRX2-dependent vascular hyperpermeability, murine DRG cells were stimulated with Der p extract, PSMα3, or capsaicin as controls. As previously reported (27, 29), all stimuli induced the release of SP from DRG cells (**Figure 7A**). Notably, the release of SP was abrogated by treatment with the TRPV1 antagonist AMG517 (**Figure 7B**), whereas it was not inhibited by treatment with the protease-activated receptor 1 (PAR1) antagonist RWJ-56110 or the PAR2 antagonist AZ3451 (**Supplementary Figure S8A**). Additionally, stimulation with mMCPT1, mMCPT4, and mMCPT6, but not with histamine, induced the release of SP from DRG cells (**Figure 7C**). We confirmed that treatment with TY-51469 inhibited the release of SP induced by mMCPT1 or mMCPT4, although it weakly inhibited that by mMCPT6 (**Figure 7D**). mMCPT4-stimulated SP release from DRG cells was not suppressed by treatment with RWJ-56110 or AZ3451 (**Supplementary Figure S8B**). Notably, intradermal injection of mMCPT4 resulted in the highest amount of extravasated dye in the ear skin of MRGPRX2-KI mice, which was lowered by treatment with anti-SP serum (**Figures 7E, F**). Moreover, PSMα3-induced MRGPRX2-dependent vascular hyper permeability was suppressed by AMG517 (**Figure 7G**). Collectively, these results indicated that PSMα3 stimulates the release of SP from TRPV1^+^ DRG cells via unknown mechanisms, subsequently inducing the degranulation of histamine and chymase via MRGPRX2, leading to vascular hyperpermeability in MRGPRX2-KI mice.

## Discussion

Various cationic drugs cause mast cell degranulation by directly activating Mrgprb2 in mice and MRGPRX2 in humans, resulting in pseudo-allergic reactions. Putative ligands for Mrgprb2 and MRGPRX2 have mainly been identified using *in vitro* assays, including mast cell degranulation assays. If intradermal administration of its putative ligand significantly increases vascular permeability in WT mice, but not in Mrgprb2-KO mice, we can conclude that it is a physiological ligand for Mrgprb2 (5–8). However, it has been difficult to accurately identify a ligand for MRGPRX2 and evaluate its *in vivo* function, because of the lack of animal models. To solve this problem, we generated MRGPRX2-KI or Mrgprb2-KO mice in which MRGPRX2 or tdTomato was expressed instead of Mrgprb2, respectively. Consistent with previous studies (5–12), surface MRGPRX2 was expressed in CTMC from MRGPRX2-KI mice. Additionally, tdTomato was highly expressed in CTMC of Mrgprb2-KO mice. However, detectable levels of surface MRGPRX2 or tdTomato were not expressed in MMC from MRGPRX2-KI or Mrgprb2-KO mice, respectively. Interestingly, Mrgprb2-KO BMMCs expressed considerable levels of tdTomato, which were lower than those expressed in PMCs. As BMMCs from MRGPRX2-KI mice failed to degranulate in response to compound 48/80, the surface expression of Mrgprb2 in BMMCs seemed to be insufficient to induce Mrgprb2-mediated degranulation.

To evaluate the degree of PMC degranulation, we stimulated PMCs from WT, Mrgprb2-KO, and MRGPRX2-KI mice with the putative ligands. Additionally, we evaluated the degree of local vascular permeability induced by the intradermal injection of the same molecule into the three types of mice. Our *in vitro* and *in vivo* results using known cationic ligands demonstrated that the degree to which a particular cationic ligand promoted vascular permeability via Mrgprb2 or MRGPRX2 in mice was proportional to its ability to induce PMC degranulation, which was largely consistent with the previously reported EC_50_ values for Mrgprb2 versus MRGPRX2 (5–8). In fact, several ligands, including compound 48/80, ciprofloxacin, and SP, increased vascular permeability in MRGPRX2-KI mice compared to WT mice. Thus, we established methods to evaluate the ligand sensitivity of MRGPRX2 *in vitro* and *in vivo*. This method may be useful for identifying novel MRGPRX2 ligands that do not act as Mrgprrb2 ligands. Alternatively, we found that Der p extract or PSMα3 did not directly activate MRGPRX2 in PMCs but induced MRGPRX2-dependent vascular hyperpermeability in mice, indicating that MRGPRX2 is indirectly activated by an endogenous ligand in these settings. One plausible explanation is that Der p extract or PSMα3 stimulates the release of SP form sensory nerve endings, which induces MRGPRX2-dependent degranulation of CTMC, because Dermatophagoides farinae was reported to directly activate TRPV1^+^ DRG cells to release SP through a cysteine protease-dependent manner (27). As a matter of fact, we found that Der p extract or PSMα3 stimulated the release of SP from murine DRG cells (27, 29); however, this release was not suppressed by treatment with a PAR1 or PAR2 inhibitor, but suppressed by that with a TRPV1 antagonist. Importantly, treatment with a TRPV1 antagonist inhibited PSMα3-induced vascular hyperpermeability, suggesting that TRPV1^+^ neurons were involved in this process. Nevertheless, the mechanisms by which Der p extract or PSMα3 activated TRPV1^+^ neurons remain to be investigated.

Notably, SP strongly stimulated the degranulation of MRGPRX2-KI PMCs to release large amounts of amines (histamine and serotonin) and proteases (tryptase and chymase), although it was reported that Mrgprb2 activation induces the release of fewer amines and more proteases than FcεRI activation (26). Considering our finding that SP strongly induces the release of histamine and chymase in human LAD2 cells, it is possible to speculate that a strong ligand, whether endogenous or exogenous, activates MRGPRX2 to induce mast cell degranulation, resulting in vascular hyperpermeability by previously unappreciated mechanisms. Importantly, treatment with an antihistamine, but not with a chymase inhibitor or anti-SP serum, inhibited FcεRI-dependent vascular hyperpermeability, whereas treatment with an antihistamine, chymase inhibitor, or anti-SP serum suppressed ciprofloxacin-, Der p extract-, or PSMα3-induced MRGPRX2-dependent vascular hyperpermeability. Additionally, treatment with an antihistamine or a chymase inhibitor suppressed SP-induced MRGPRX2-dependent vascular hyperpermeability. Remarkably, MCPT4, but not histamine, stimulated the DRG cells to release SP, whereas intradermal injection of MCPT4 or histamine induced SP-dependent or -independent vascular hyperpermeability, respectively. Collectively, it is plausible to assume that neuronal SP-driven MRGPRX2-depdenent CTMC degranulation contributes to vascular hyperpermeability via histamine and MCPT4. Histamine directly acts on blood vessels to increase vascular permeability, whereas MCPT4 activates sensory neurons to release SP, which can further enhance vascular permeability indirectly. However, the involvement of released mast cell tryptases in sensory neuron activation under such conditions cannot be ruled out. Considering the multiple functions of mast cell chymases and tryptases into consideration (13, 14, 38–43), further examination is necessary to completely understand the mechanisms by which MCPTs activate the sensory neurons.

Previous studies on pseudo-allergy models using WT and Mrgprb2 mutant mice showed that several cationic drugs cause CTMC degranulation via Mrgprb2 to directly increase vascular permeability (5). On the other hand, our results using MRGPRX2-KI mice showed that the same drugs degranulate CTMC via MRGPRX2 to increase vascular permeability directly and indirectly via DRG cell activation, where neuronal SP amplifies CTMC degranulation via MRGPRX2. This is presumably due to larger amounts of histamine and chymase released by MRGPRX2-stimulated CTMC as compared with Mrgprb2 stimulated-CTMC. One plausible reason is that MRGPRX2 shows a higher affinity to SP than Mrgprb2 does (5). Another possibility is that surface expression levels of MRGPRX2 or signaling pathways downstream of MRGPRX2 may be somehow different from those of Mrgprb2. Further examination will be required to solve this question. Importantly, we provided evidence that CTMC degranulation product chymase activates DRG cells to release SP, which in turn induces CTMC degranulation via MRGPRX2. Thus, the worsening cycle (MRGPRX2 → CTMC degranulation → chymase → DRG activation → SP → MRGPRX2) contributes to vascular hyperpermeability in MRGPRX2-KI mice in pseudo-allergy models, which is a novel point. In any case, our studies clarified the possible involvement of neuronal SP in human pseudo-allergy. Moreover, we showed that the worsening cycle (DRG activation → SP → MRGPRX2 → CTMC degranulation → chymase → DRG activation) also contributes to vascular hyperpermeability in MRGPRX2-KI mic in response to intradermal injection of Der p or PSMα3, although it was expected that Der p or PSMα3 activates DRG cells to release SP (27, 29). These novel findings will help understand the roles of MRGPRX2-mediated mast cell degranulation in human vascular permeability. In addition, our conclusion was drawn by analyzing MRGPRX2-KI mice (versus WT and Mrgprb2-KO mice). Although we must recognize the pros and cons in the analysis of KO and KI mice, *in vitro* and *in vivo* experiments using these mice will be useful to analyze MRGPRX2 ligands and their functions.

In conclusion, our results revealed that neuronal SP-driven MRGPRX2-depdendent mast cell degranulation products, histamine and chymase, differentially promoted vascular permeability, suggesting the roles of MC_TC_-expressing MRGPRX2 in human inflammatory diseases. Further analysis of MRGPRX2-KI mice is necessary to understand the mechanisms underlying IgE-independent inflammation, pain, and itching in humans.

## Data Availability

The original contributions presented in the study are included in the article/**Supplementary Material**. Further inquiries can be directed to the corresponding authors.

## Glossary

Abs: antibodies
Ag: antigen
ANOVA: analysis of variance
APC: allophycocyanin
BMMC: bone marrow-derive mast cell
BSA: bovine serum albumin
BV421: Brilliant Violet 421
CTMC: connective tissue mast cell
Cy: cyanine
Der p: Dermatophagoides pteronyssinus
DMEM: Dulbeccos Modification of Eagles Medium
DNP: 2,4-dinitrophenyl
DRG: dorsal root ganglion
EC_50_: 50% effective concentration
EDTA: ethylene diamine tetra acetic acid
ELISA: enzyme-linked immuno-sorbent assay
EpCAM: epithelial cell adhesion molecule
FBS: fetal bovine serum
FCS: fetal calf serum
FcεRI: high-affinity immunoglobulin E receptor
FITC: fluorescein isothiocyanate
GDNF: glial cell-line derived neurotrophic factor
HDM: house dust mite
HBSS: Hanks’ balanced salt solution
HAS: human serum albumin
IL: interleukin
IMDM: Iscove’s modified Dulbecco medium
IgE: immunoglobulin E
mMCPT: mouse mast cell protease
MC_T_: tryptase-expressing mast cell
MC_TC_: tryptase- and chymase-expressing mast cell
MMC: mucosal mast cell
Mrgprb2-KO: Mrgprb2 knockout
MRGPR: Mas-related G protein-coupled receptor
MRGPRX2-KI: MRGPRX2 knock-in
NGF: nerve growth factor
PAR: protease activated receptor
PBS: phosphate-buffered saline
PCR: polymerase chain reaction
PE: phycoerythrin
PMCs: peritoneal mast cells
PSMα3: phenol-soluble modulin α3
RPMI: Roswell Park Memorial Institute
SCF: stem cell factor
SD: standard deviation
SFM: serum-free media
SP: substance P
TNP: 2,4,6-trinitrophenyl
TRPV1: transient receptor potential vanilloid 1
WT: wild-type
