# Supplementary material for: Neuronal substance P-driven MRGPRX2-dependent mast cell degranulation products differentially promote vascular permeability
Source: Front Immunol. 2024 Nov 21;15:1477072. doi: 10.3389/fimmu.2024.1477072 (PMC11617324; doi:10.3389/fimmu.2024.1477072)

frontiers

Supplementary Material

Neuronal substance P-driven MRGPRX2-dependent mast cell degranulation products differentially promote vascular permeability

Masakazu Nagamine, Ayako Kaitani*, Kumi Izawa, Tomoaki Ando, Akihisa Yoshikawa, Masahiro Nakamura, Akie Maehara, Risa Yamamoto, Yoko Okamoto, Hexing Wang, Hiromichi Yamada, Keiko Maeda, Nobuhiro Nakano, Toshiaki Shimizu, Hideoki Ogawa, Ko Okumura, Jiro Kitaura*

*** Correspondence:**

Ayako Kaitani
a-kaitani@juntendo.ac.jp

Jiro Kitaura
j-kitaura@juntendo.ac.jp

SUPPLEMENTARY FIGURE LEGENDS

Supplementary Figure 1. Generation of MRGPRX2-KI and Mrgprb2-KO mice. (**A**) Strategy for the establishment of MRGPRX2-KI and Mrgprb2-KO mouse lines. Schematic representation of *Mrgprb2* alle, targeting vector, targeted allele, Flp-mediated allele, and Cre-mediated allele. Black box indicates the exon. Yellow box indicates the *Mrgprb2* coding region. MC1_DT-A is the diphtheria toxin A fragment gene directed by the MC1 promoter for the negative selection of homologous recombinants. PGK neo, neomycin-resistant gene directed by the PGK gene promoter, is flanked by FRT sequences at the 5'- and 3'- ends. Black and white triangles indicate the loxP and FRT sequence, respectively. Red and blue boxes indicate the tdTomato and MRGPRX2 coding region, respectively. White box indicates the bovine growth hormone polyadenylation sequence, BGH pA. Mrgprb2-KO allele was generated from the targeted allele via Flp-mediated deletion, whereas MRGPRX2-KI allele was generated from Mrgprb2-KO allele via Cre-mediated deletion. (**B**) Relative mRNA expression levels of *Mrgprb2*, *MRGPRX2*, and *tdTomato* in the PMCs from WT, Mrgprb2-KO (b2-KO), or MRGPRX2-KI (X2-KI) mice and DRG cells from WT mice.

Supplementary Figure 2. Percentages of β-hexosaminidase released by PMCs in response to compound 48/80 are highest in MRGPRX2-KI PMCs. Percentages of β-hexosaminidase released by WT, Mrgprb2-KO (b2-KO), and MRGPRX2-KI (X2-KI) PMCs after treatment with the indicated concentrations of compound 48/80 for 30 min. Data are representative of three independent experiments and indicate the mean ± SD. $^{*}P < 0.05$ and $^{**}P < 0.01$.

Supplementary Figure 3. Expression levels of MRGPRX2 and tdTomato in the PMCs from WT, Mrgprb2-KO, and MRGPRX2-KI mice with BALB/c background. Surface expression levels of FcεRIα and c-Kit (upper panel), expression levels of tdTomato (middle panel), and surface expression levels of MRGPRX2 (lower panel) in the PMCs from WT, Mrgprb2-KO (b2-KO), or MRGPRX2-KI (X2-KI) mice with BALB/c background. Data are representative of three independent experiments.

Supplementary Figure 4. LAD2 cells release higher amounts of histamine and chymase in response to SP compared to that with FcεRI crosslinking. (**A-C**) Percentages of surface $CD63^+$ LAD2 cells (**A**) and levels of histamine (**B**) and chymase (**C**) in the culture supernatants of ant-TNP IgE-sensitized LAD2 cells after treatment with the indicated concentrations of TNP-BSA and SP. Data are representative of three independent experiments and indicate the mean ± SD. **P* < 0.05 and ***P* < 0.01.

Supplementary Figure 5. MRGPRX2-KI PMCs release larger amounts of LTB4 and cysteinyl LT in response to SP than the WT PMCs. (**A**, **B**) Levels of LTB4 (**A**) and cysteinyl LT (**B**) in the culture supernatants of WT, Mrgprb2-KO (b2-KO), and MRGPRX2-KI (X2-KI) PMCs after treatment with the indicated concentrations of SP for 1 h. Data are representative of three independent experiments and indicate the mean ± SD. **P* < 0.05 and ***P* < 0.01.

Supplementary Figure 6. A MRGPRX2 antagonist suppresses SP- or compound 48/80-stimulated degranulation in MRGPRX2-KI PMCs. Percentages of surface $CD63^+$ MRGPRX2-KI PMCs after treatment with the indicated concentrations of SP or compound 48/80 in the presence of 200 μM Piperine or vehicle. Data are representative of two independent experiments and indicate the mean ± SD. ***P* < 0.01

Supplementary Figure 7. Anti-SP serum treatment does not suppress histamine-induced vascular hyperpermeability in WT mice. Quantification of the Evans blue dye that extravasated into the ear skin in WT mice intradermally injected with 100 μg histamine along with 15 μL of anti-SP serum or control serum. Data are representative of two independent experiments. n = 4; ± SD.

Supplementary Figure 8. Treatment with the PAR1 and PAR2 antagonists does not suppress the release of SP from DRG cells stimulated by Der p extract, PSMα3, and mMCPT4. (**A, B**) Levels of SP in the culture supernatants of murine DRG cells. DRG cells were stimulated with 30 μg/mL Der p

extract, 30 μg/mL PSMα3, and PBS (**A**) or 5 μg/mL mMCPT4 and PBS (**B**) in the presence of 10 μM RWJ-56110, 10 μM AZ3451, and vehicle. Data are representative of three independent experiments and indicate the mean ± SD.

SUPPLEMENTARY FIGURE 1

A

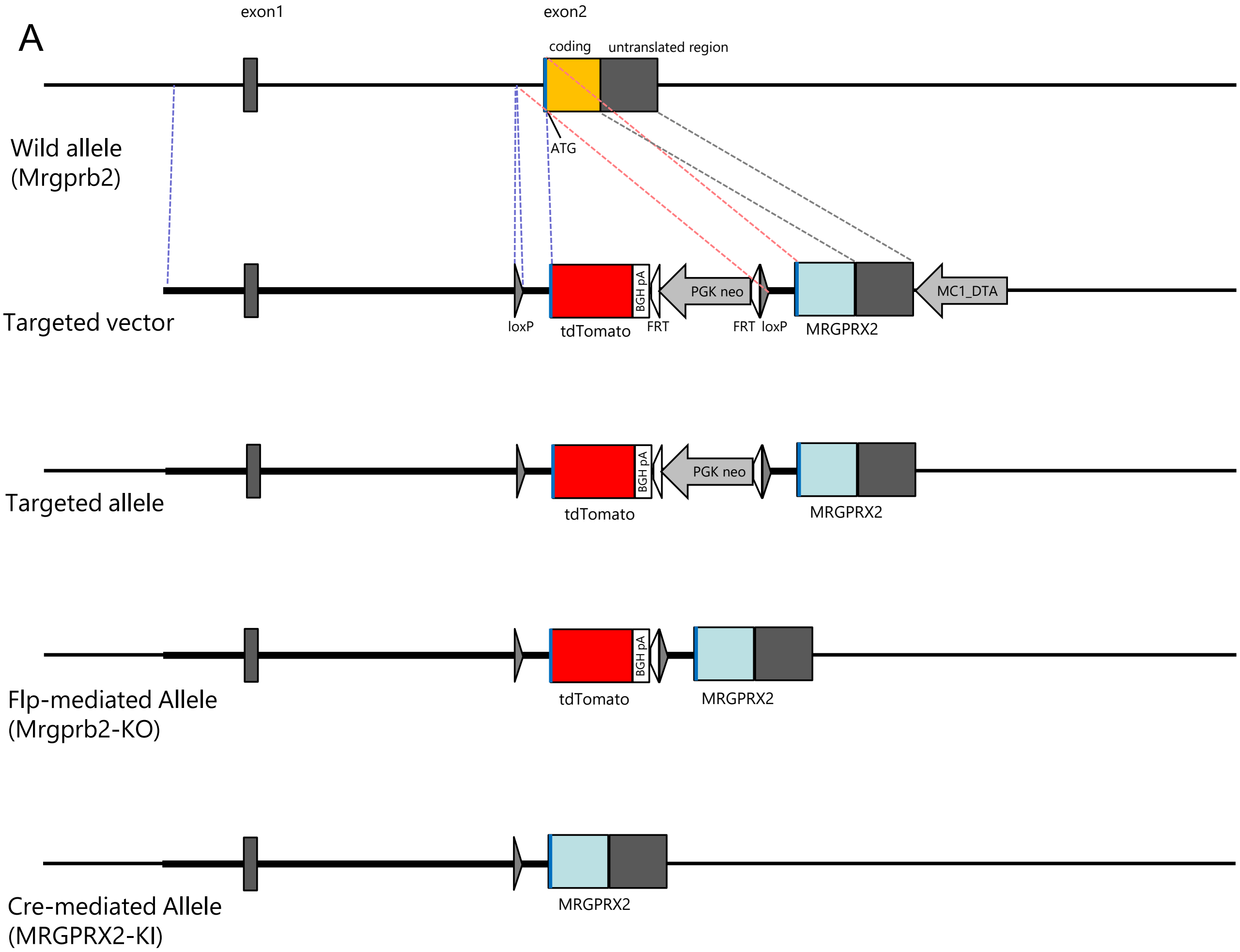

B

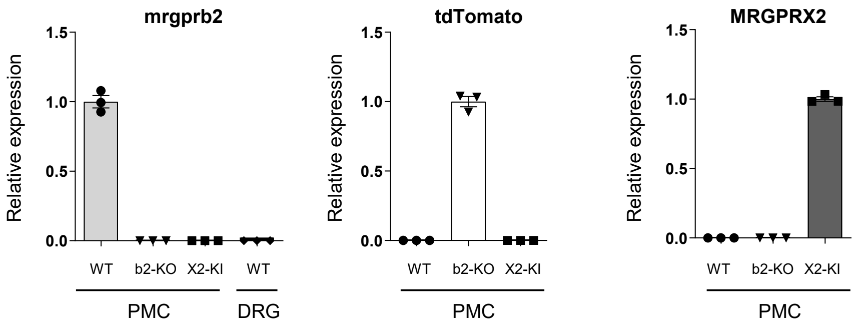

SUPPLEMENTARY FIGURE 2

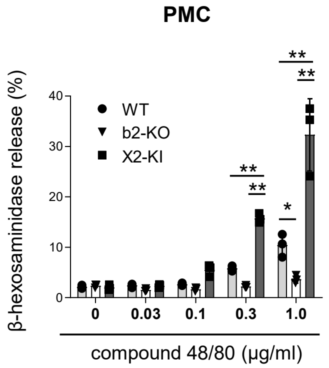

SUPPLEMENTARY FIGURE 3

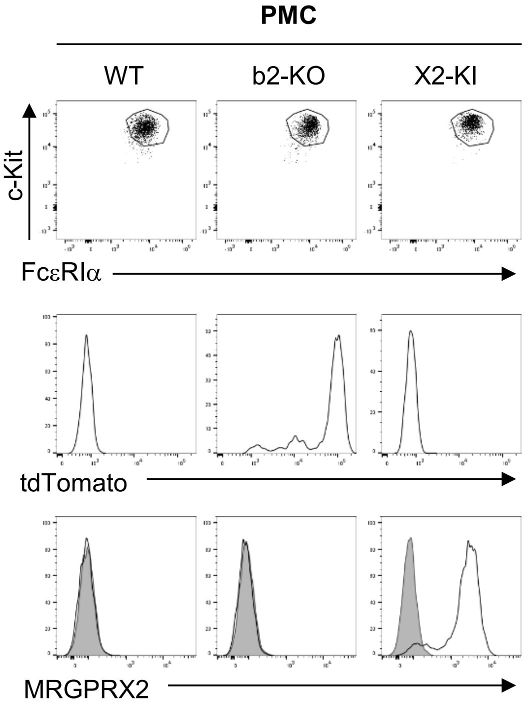

SUPPLEMENTARY FIGURE 4

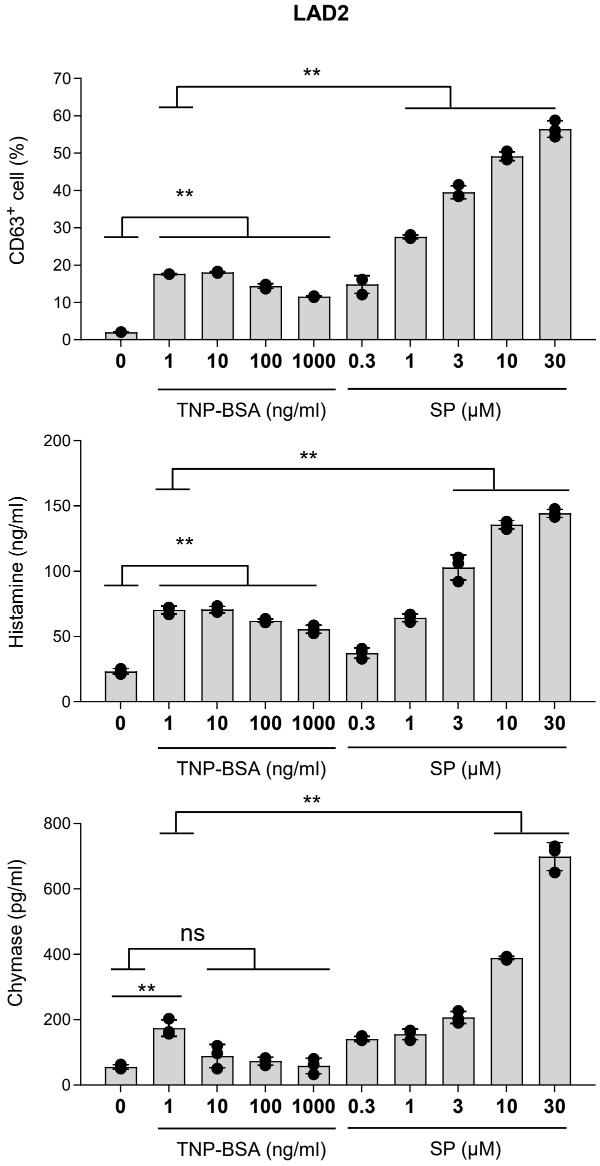

SUPPLEMENTARY FIGURE 5

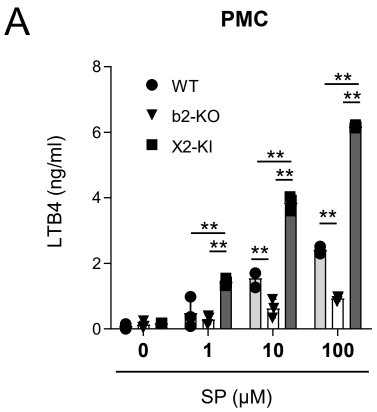

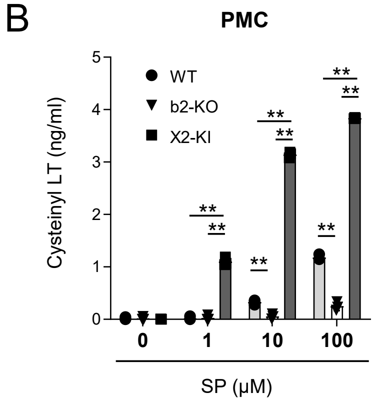

SUPPLEMENTARY FIGURE 6

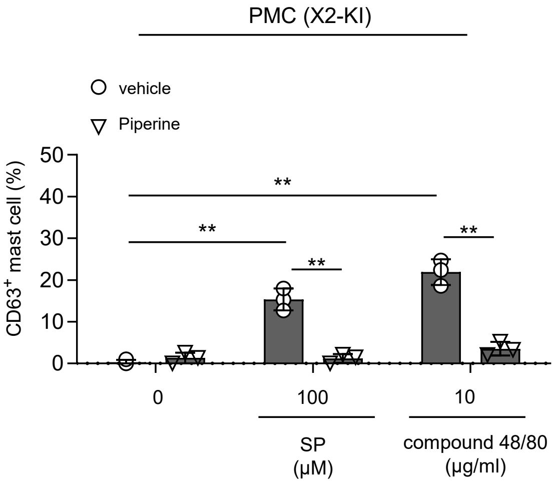

SUPPLEMENTARY FIGURE 7

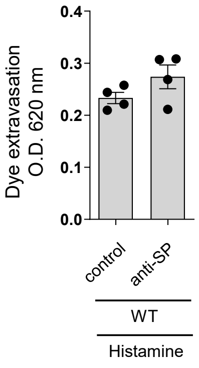

SUPPLEMENTARY FIGURE 8

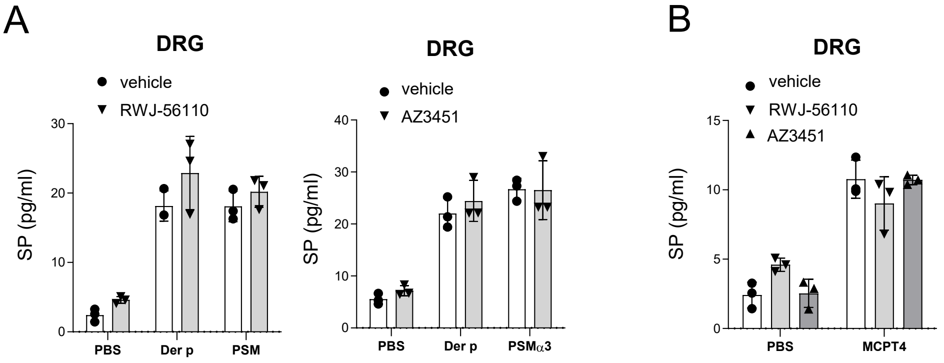

Supplement: Supplementary file 1 [file DataSheet1.pdf]
